# Supplementary material for: Sex-associated molecular differences for cancer immunotherapy
Source: Nat Commun. 2020 Apr 14;11:1779. doi: 10.1038/s41467-020-15679-x (PMC7156379; doi:10.1038/s41467-020-15679-x)
Supplement: Supplementary file 1 — Supplementary Information [file 41467_2020_15679_MOESM1_ESM.pdf]

Sex-associated molecular differences for cancer immunotherapy

Youqiong Ye, Ying Jing, Liang Li, Gordon B. Mills, Lixia Diao*, Hong Liu*, Leng Han*

Supplementary Information

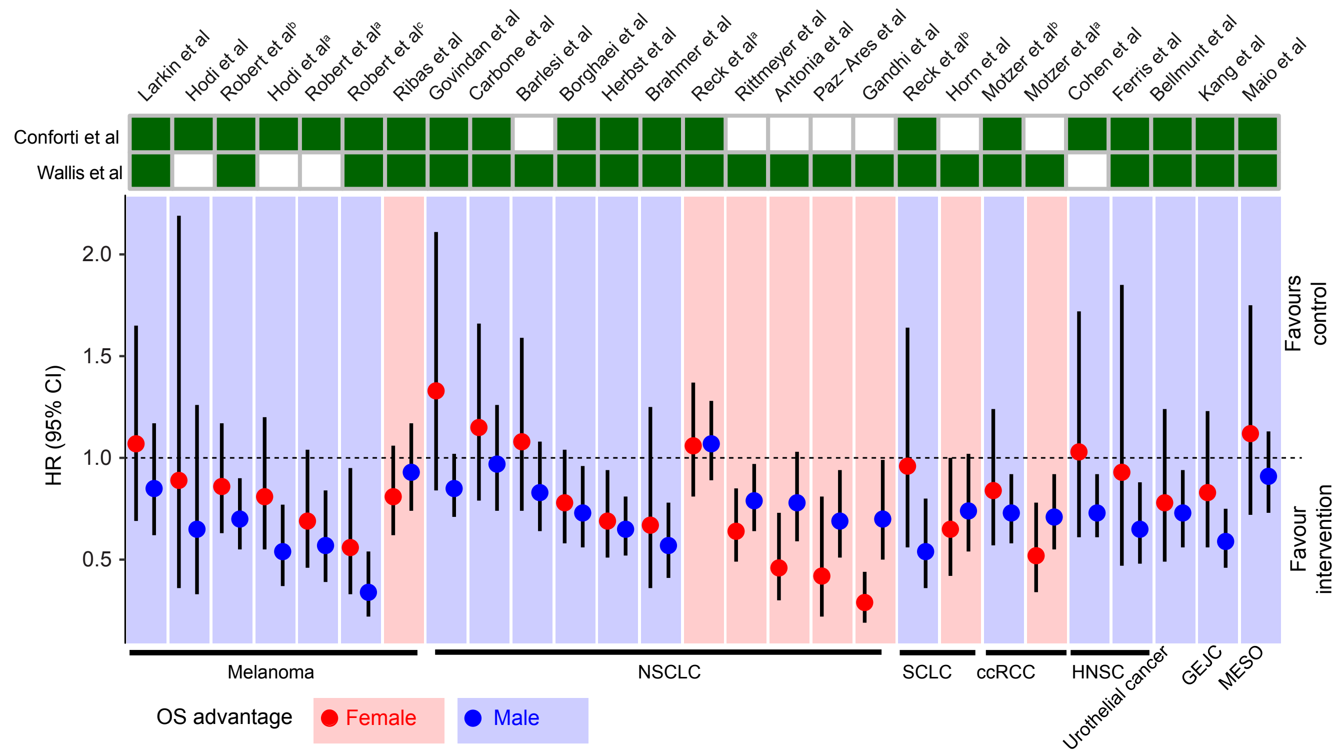

Supplementary Fig. 1 Summary of overall survival in meta-analysis. Hazard ratios (HR) of overall survival for female and male patients assigned to intervention treatment (immunotherapy: IO), compared with those assigned to control treatment in 27 trials from Conforti et al. and Wallis et al.. The dots represent trial-specific HRs. Horizontal lines indicate the 95% CIs. Background color indicates OS advantage of ICB treatment in female (red) or male (blue). The sample size of female and male patients are deposited in supplementary table 1.Abbreviation: ccRCC, clear cell renal cell carcinoma; HNSC, head and neck cancer; NSCLC, Non-Small Cell Lung Cancer; SCLC, Small Cell Lung Cancer; GEJC, Gastric or gastroesophageal junction carcinoma; MESO, Mesothelioma.

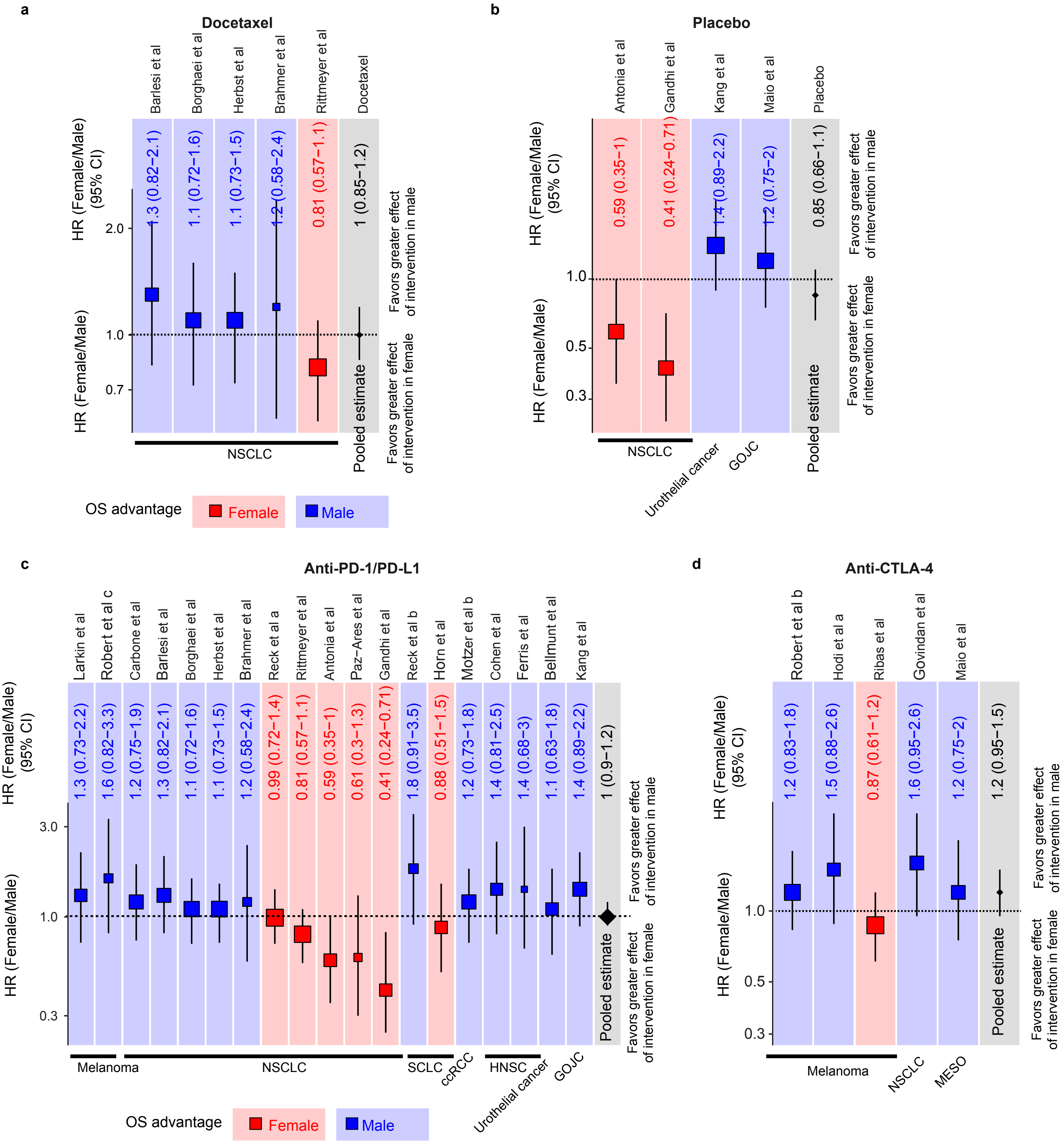

Supplementary Fig. 2 Clinical outcome between male and female patients with immunotherapy. (**a-b**) The association of immunotherapy outcome and gender in immunotherapy vs different control arm, including Docetaxel treatment (**a**) and Placebo treatment (**b**). (**c-d**) The association of immunotherapy outcome and gender in anti-PD-1/PD-L1 (**c**) and anti-CTLA-4 (**d**) therapies. Background and square color indicate OS advantage of ICB treatment in female (red) or male (blue). Square size indicates the proportion to the inverse of the variance of the estimates. Black vertical lines indicate the 95% Confidence Interval (CI). The dots represent trial-specific HRs. Horizontal lines indicate the 95% CIs. Background color indicates OS advantage of ICB treatment in female (red) or male (blue). The sample size of female and male patients are deposited in supplementary table 1. Abbreviation: ccRCC, clear cell renal cell carcinoma; HNSC, head and neck cancer; NSCLC, Non-Small Cell Lung Cancer; SCLC, Small Cell Lung Cancer; GEJC, Gastric or gastroesophageal junction carcinoma; MESO, Mesothelioma.

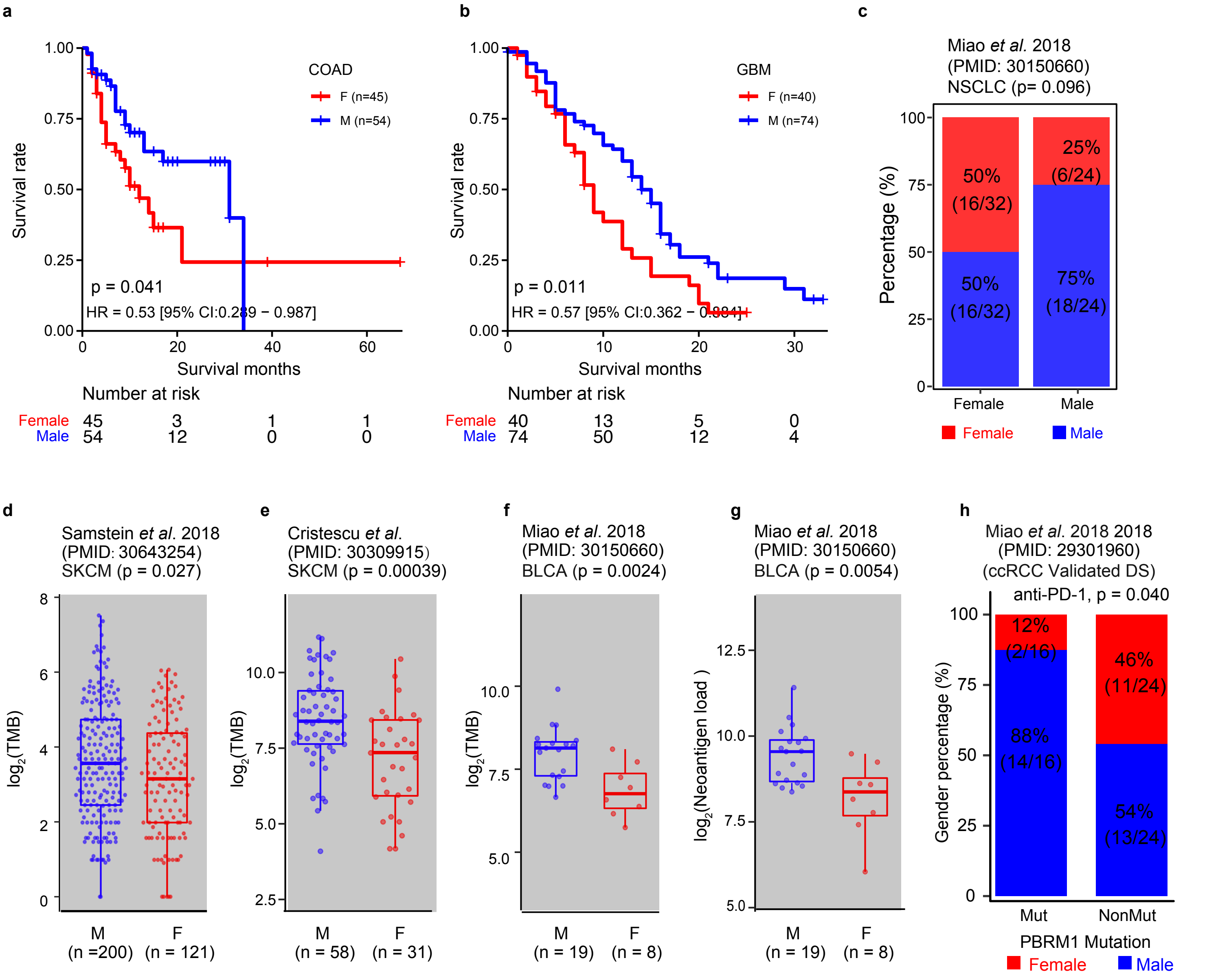

Supplementary Fig. 3 Overall survival, response, and molecular differences in male and female patients with ICB treatment. (**a-b**) Kaplan-Meier curves show overall survival for male and female patients with COAD (**a**, female n = 45 and male n = 54) and GBM (**b**, female n = 40 and male n = 74) under anti-PD1 treatment from dataset PMID: 30643254. A two-sided log-rank test $P < 0.05$ is considered as a statistically significant difference. (**c**) The percentage (%) of benefit (red) and non-benefit (blue) in female (n = 32) and male (n = 24) patients with NSCLC after ICB treatment. (**d-f**) Differences in TMB between male and female patients with SKCM in datasets (**d**) PMID: 30643254 and (**e**) PMID: 30309915; and in patients with BLCA in dataset (**f**) PMID: 30150660. (**g**) Difference in the neoantigen load in dataset PMID: 30150660. (**h**) Difference in the percentage (%) of renal cell carcinoma with or without PBRM1 mutation between male and female patients in dataset PMID: 30150660. Boxes in **d-g** indicate the median ±1 quartile, with whiskers extending from the hinge to the smallest or largest value within 1.5 interquartile range from the box boundaries. Sample size is labeled under X-axis. P values in **d-g** were determined by two-sided Wilcoxon–Mann–Whitney test. P value in **c** and **h** were determined by two-sided Fisher's exact test.

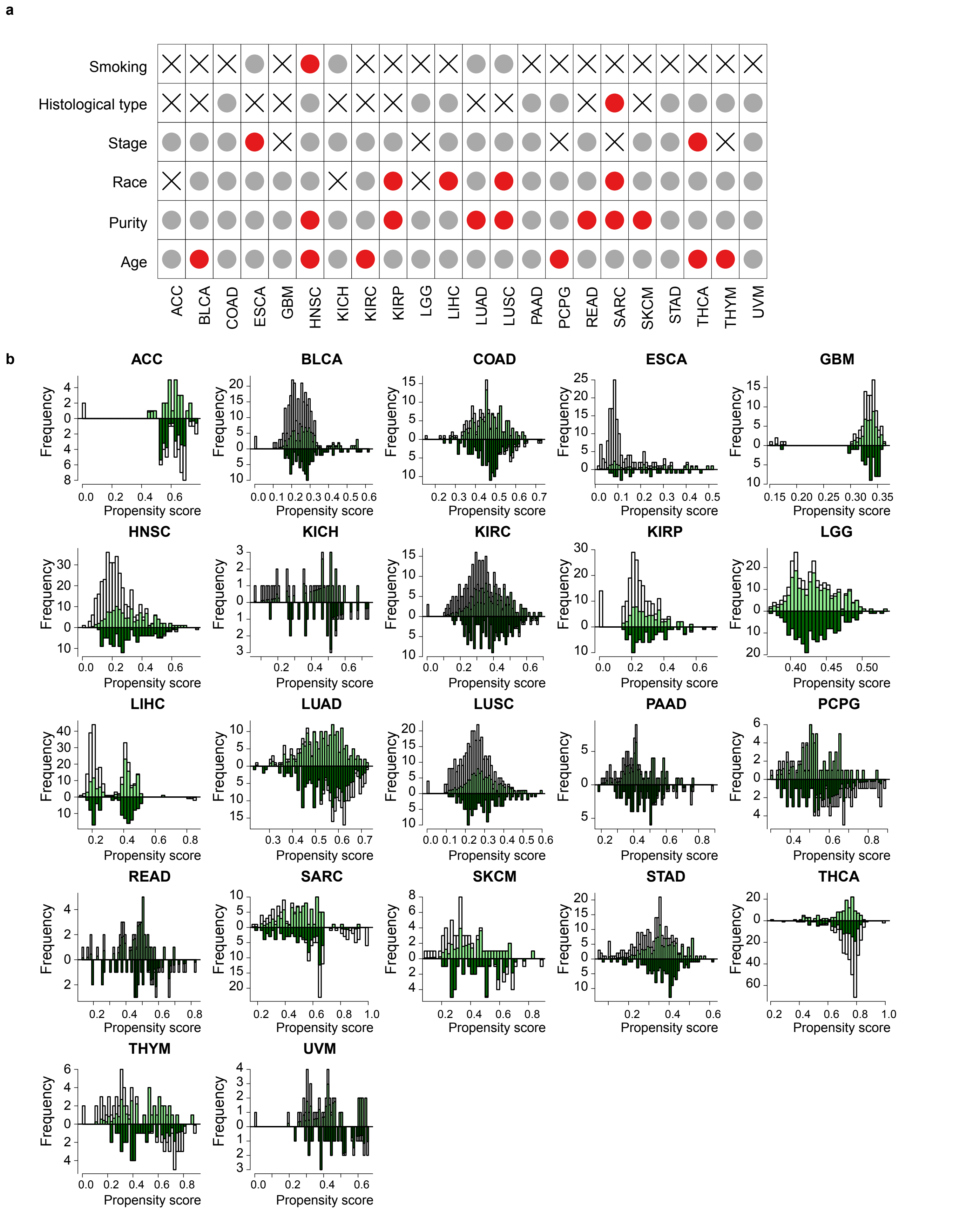

Supplementary Fig. 4 Potential confounding factors and propensity score between female and male patients across 22 cancer types. (**a**) Heatmap shows p-values (two-sided Wilcoxon–Mann–Whitney test for continuous variables, including age at diagnosis and tumor purity, and Fisher's exact test for discrete variables, including race, tumor stage, histological type, and smoking history) with significance (red dot) or non-significance (gray dot; using p = 0.05 as the cutoff). Features marked as X indicate data not available. (**b**) A mirror histogram of overall the range of propensity score from the female patients coincides with that of male patients. Sample size for each dataset was listed in Supplementary Table 3.

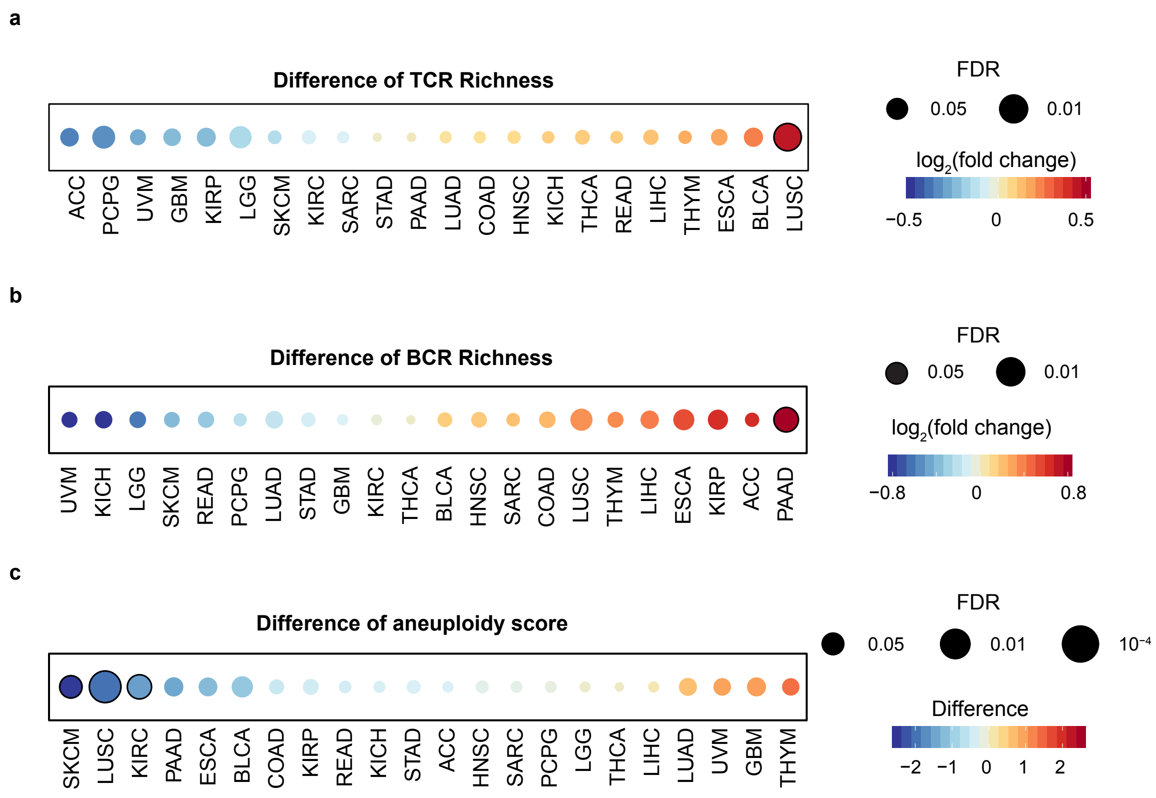

Supplementary Fig. 5 Differences of TCR/BCR richness, and aneuploidy score between male and female patients across cancer types. Differences in (**a**) TCR richness, (**b**) BCR richness, and (**c**) aneuploidy score across cancer types. Statistical analysis was performed using propensity score algorithm (see online methods) to identify gender-associated TCR/BCR richness and aneuploidy score. Sample size for each dataset was listed in supplementary table 3.

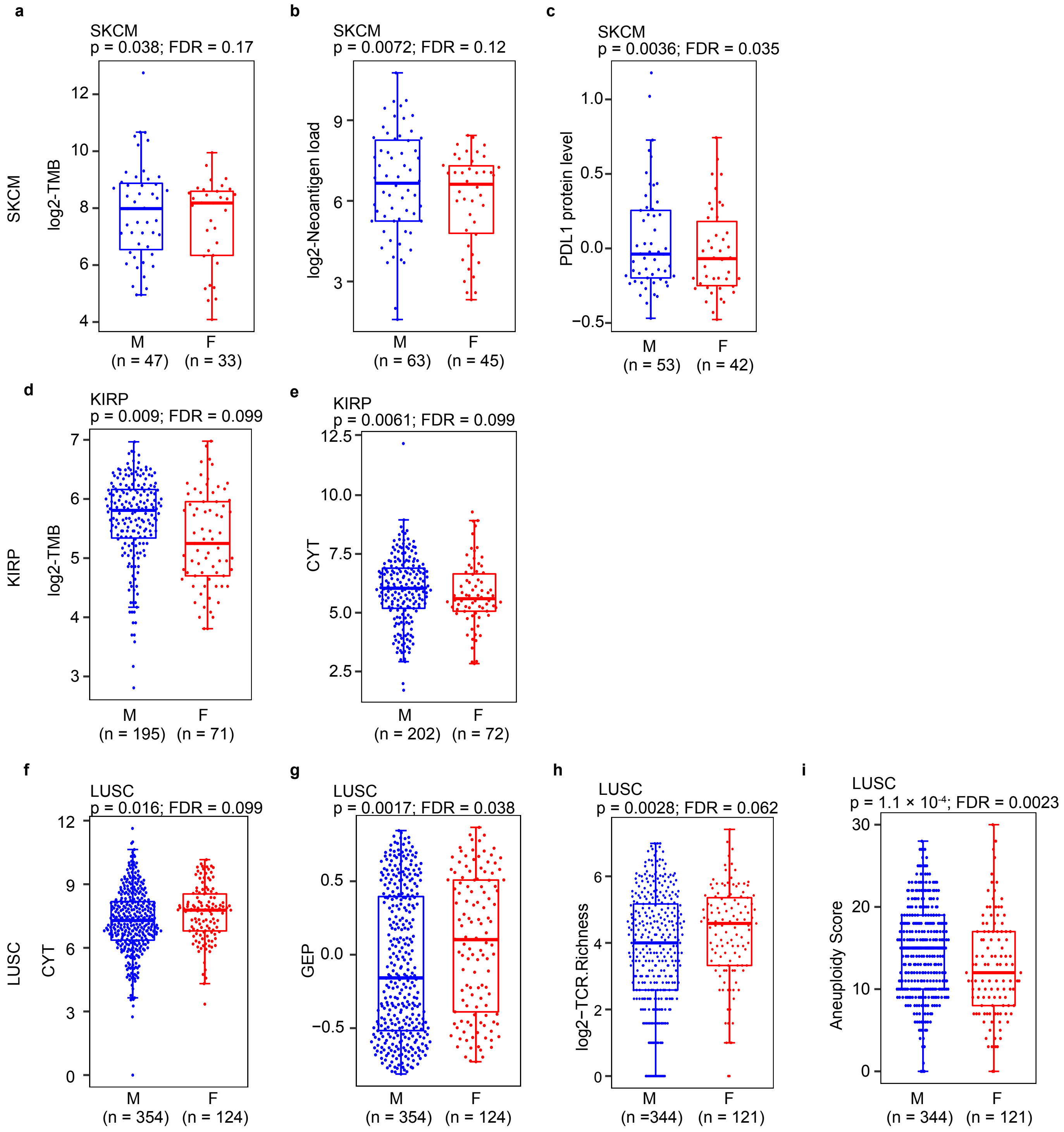

Supplementary Fig. 6 Molecular differences in male and female patients from TCGA. (**a**) TMB, (**b**) neoantigen load, and (**c**) PD-L1 protein expression between male and female patients with SKCM. (**d**) TMB and (**e**) CYT between male and female patients with KIRP. (**f**) CYT, (**g**) GEP, (**h**) TCR richness, and (**i**) aneuploidy score between male and female patients with LUSC. The boxes indicate the median ±1 quartile, with whiskers extending from the hinge to the smallest or largest value within 1.5 interquartile range from the box boundaries. Statistical analysis was performed using propensity score algorithm to identify gender-associated immune features; p-value was calculated by linear regression model and adjusted by Benjamini & Hochberg correction; details are in Methods. Sample size is labeled under X-axis.

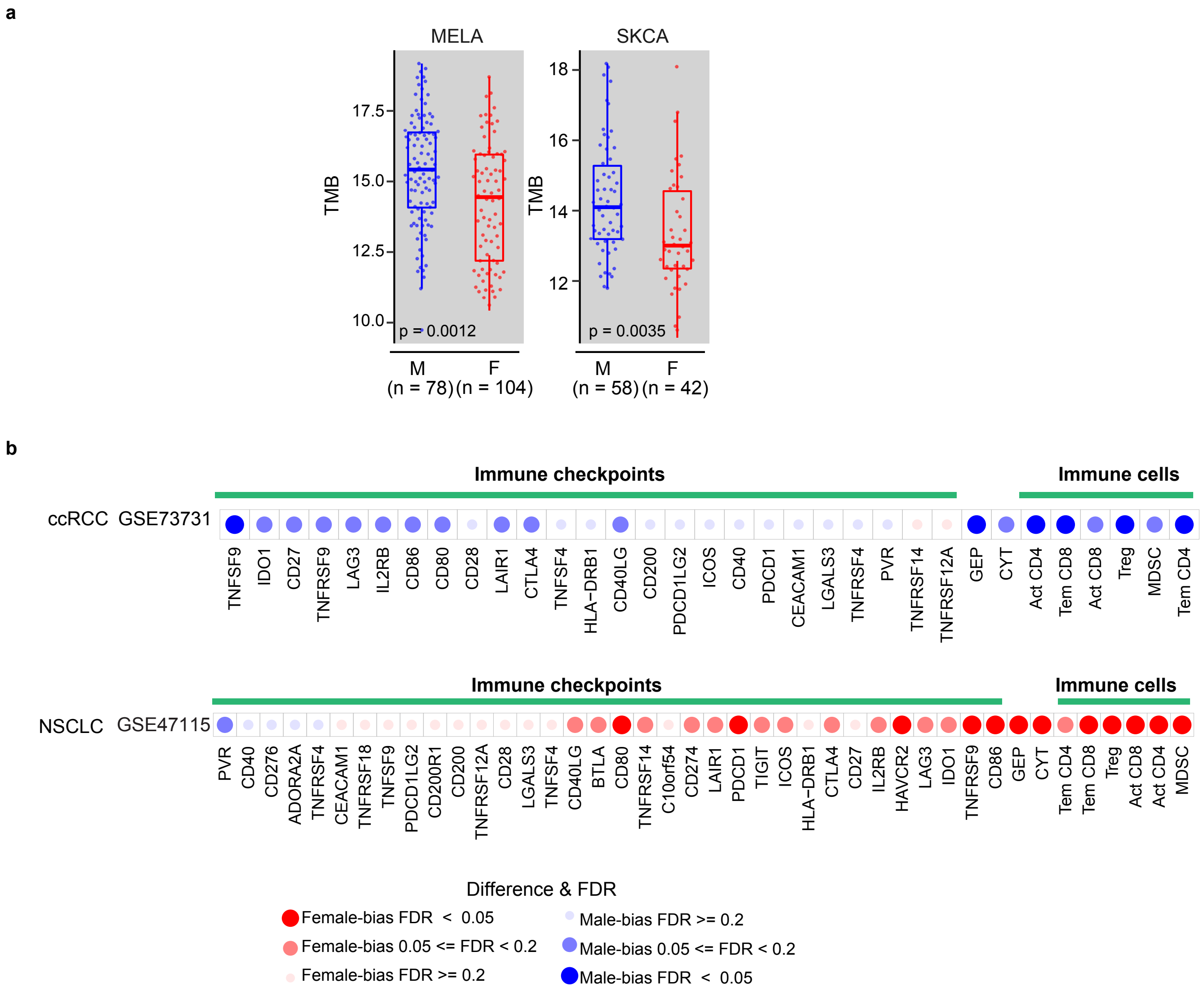

Supplementary Fig. 7 Molecular differences in male and female patients in independent datasets. (**a**) Higher TMB in male patients with melanoma in two independent datasets, Australia (Skin Cancer - Australia [MELA-AU]) and Brazil (Skin Adenocarcinoma – Brazil [SKCA-BR]) through the International Cancer Genome Consortium project. The boxes indicate the median ±1 quartile, with whiskers extending from the hinge to the smallest or largest value within 1.5 interquartile range from the box boundaries. Sample size is labeled under X-axis. (**b**) Checkpoints, GEP, CYT, and relative abundance of immune cell populations between male and female patients with ccRCC (GSE73731, female n = 102, male n = 163) and NSCLC (GSE47115, female n = 21, male n = 25). Statistical analysis in A and B was performed using propensity score algorithm (see online methods) to identify gender-associated immune features. p-value was calculated by linear regression model and adjusted by Benjamini & Hochberg correction.

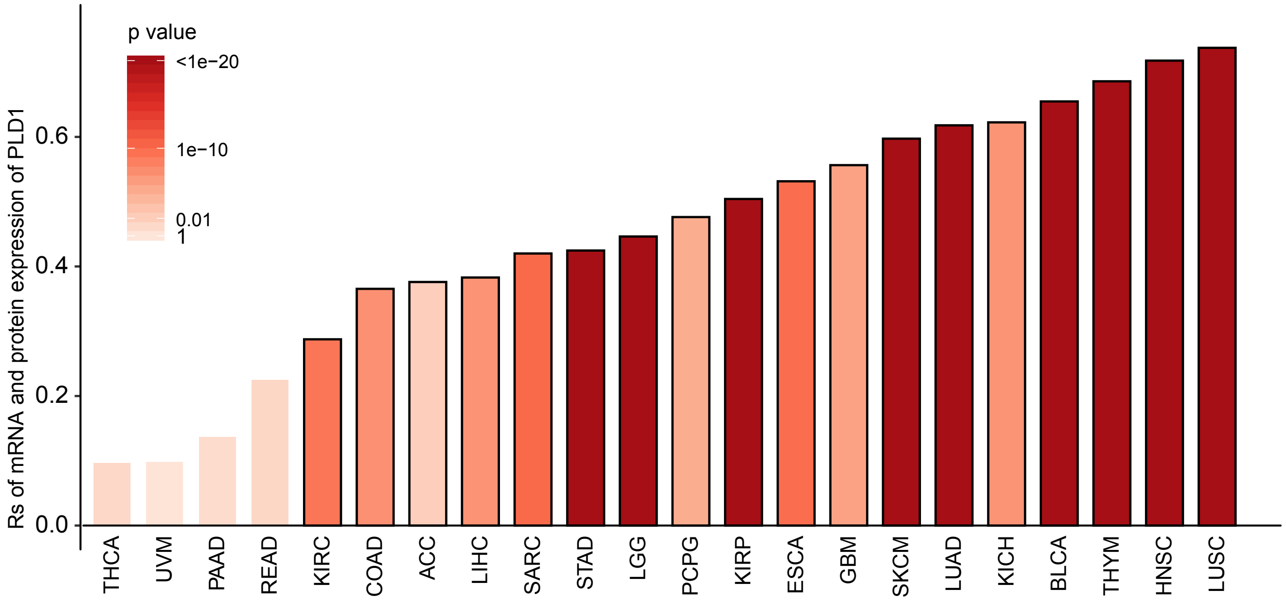

Supplementary Fig. 8 The Spearman correlation between protein level and mRNA expression of PD-L1 across 22 cancer types. The Spearman correlation coefficient (*Rs*) and p-value was performed by Spearman correlation. The black border indicates $p < 0.05$. Sample size was listed in supplementary table 3.

Supplementary Tables

Supplementary Table 1. Characteristics and outcomes of 27 trials included from two meta-analysis studies with ICB treatment (Conforti et al[4] and Wallis et al[3]).

Dataset (PubMed ID/Refs)	Author, Year	ClinicalTrials.govID	Cancer type	Intervention (No.)	Control Treatment (No.)	Sex, No.		Overall Survival HR (95% CI)		Conforti et al	Wallis et al
						F	M	F	M		
30280658[47]	Antonia *et al*. 2018	NCT02395172	NSCLC	Durvalumab (476)	Placebo (237)	213	500	0.46 (0.30-0.73)	0.78 (0.59-1.03)	No	Yes
30262187[48]	Barlesi *et al*. 2018	NCT02395172	NSCLC	Avelumab (396)	Docetaxel (396)	162	367	1.08 (0.74-1.59)	0.83 (0.64-1.08)	No	Yes
28212060[49]	Bellmunt *et al.*2017	NCT02256436	Urothelial	Pembrolizumab (270)	ICC (272)	140	402	0.78 (0.49-1.24)	0.73 (0.56-0.94)	Yes	Yes
26412456[50]	Borghaei *et al.*2015	NCT01673867	NSCLC	Nivolumab (292)	Docetaxel (290)	263	319	0.78 (0.58-1.04)	0.73 (0.56-0.96)	Yes	Yes
26028407[51]	Brahmer *et al.* 2015	NCT01642004	NSCLC	Nivolumab (135)	Docetaxel (137)	64	208	0.67 (0.36-1.25)	0.57 (0.41-0.78)	Yes	Yes
28636851[52]	Carbone et al	NCT02041533	NSCLC	Nivolumab (271)	ICC (270)	209	332	1.15 (0.79-1.66)	0.97 (0.74-1.26)	Yes	Yes
30509740[53]	Cohen *et al.* 2019	NCT02252042	HNSC	Pembrolizumab (n=247)	ICC (n=248)	412	495	1.03 (0.61-1.72)	0.73 (0.61-0.92)	Yes	No
27718784[54]	Ferris *et al.* 2016	NCT02105636	HNSC	Nivolumab (240)	ICC (121)	61	300	0.93 (0.47-1.85)	0.65 (0.48-0.88)	Yes	Yes
29658856[55]	Gandhi *et al.* 2018	NCT02578680	NSCLC	Pembrolizumab + platinum (410)	Placebo + platinum (206)	253	363	0.29 (0.19-0.44)	0.7 (0.50-0.99)	No	Yes
28854067[56]	Govindan *et al.*2017	NCT01285609	NSCLC	Ipilimumab + paclitaxel + carboplatin (388)	Placebo + paclitaxel + carboplatin (361)	114	635	1.33 (0.84-2.11)	0.85 (0.71-1.02)	Yes	Yes
26712084[57]	Herbst *et al.* 2016	NCT01905657	NSCLC	Pembrolizumab (346)	Docetaxel (343)	399	634	0.69 (0.51-0.94)	0.65 (0.52-0.81)	Yes	Yes
27622997[58]	Hodi *et al.* 2016	NCT01927419	Melanoma	Nivolumab plus Ipilimumab (n=95)	Ipilimumab plus placebo (n=47)	95	142	0.89 (0.36-2.19)	0.65 (0.33-1.26)	Yes	No
20525992[59]	Hodi *et al.* 2010	NCT00094653	Melanoma	Ipilimumab (n=137)	gp100 (n=136)	275	401	0.81 (0.55-1.20)	0.54 (0.37-0.77)	Yes	No
30280641[60]	Horn *et al.* 2018	NCT02763579	SCLC	Atezolizumab + carboplatin + etoposide (201)	Placebo + carboplatin + etoposide (202)	142	261	0.65 (0.42-1.00)	0.74 (0.54-1.02)	No	Yes
28993052[61]	Kang *et al.* 2017	NCT02267343	GOJC	Nivolumab (330)	Placebo (163)	145	348	0.83 (0.56-1.23)	0.59 (0.46-0.75)	Yes	Yes
28671856[62]	Larkin *et al.* 2018	NCT01721746	Melanoma	Nivolumab (272)	ICC (133)	144	261	1.07 (0.69-1.65)	0.85 (0.62-1.17)	Yes	Yes
28729154[63]	Maio *et al.*2017	NCT01843374	MESO	Tremelimumab (382)	Placebo (189)	137	434	1.12 (0.72-1.75)	0.91 (0.73-1.13)	Yes	Yes
29562145[64]	Motzer *et al.* 2018	NCT02231749	ccRCC	Nivolumab (410)	Everolimus (411)	202	619	0.52 (0.34-0.78)	0.71 (0.55-0.92)	No	Yes
26406148[65]	Motzer *et al.* 2015	NCT01668784	ccRCC	Nivolumab + ipilimumab (425)	Sunitinib (422)	232	615	0.84 (0.57-1.24)	0.73 (0.58-0.92)	Yes	Yes
30280635[66]	Paz-Ares *et al.* 2018	NCT02775435	NSCLC	Pembrolizumab + ICC (278)	Placebo + ICC (281)	104	455	0.42 (0.22-0.81)	0.69 (0.51-0.94)	No	Yes
27458307[67]	Reck *et al.* 2016	NCT01450761	NSCLC	Ipilimumab + etoposide + platinum (478)	Placebo + etoposide + platinum (476)	311	643	1.06 (0.81-1.37)	1.07 (0.89-1.28)	Yes	Yes
27718847[68]	Reck *et al.* 2016	NCT02142738	SCLC	Pembrolizumab (154)	ICC (151)	311	643	0.96 (0.56-1.64)	0.54 (0.36-0.80)	Yes	Yes
23295794[69]	Ribas *et al.* 2013	NCT00257205	Melanoma	Tremelimumab (328)	ICC (327)	283	372	0.81 (0.62-1.06)	0.93 (0.74-1.17)	Yes	Yes
27979383[70]	Rittmeyer *et al. 2017*	NCT02008227	NSCLC	Atezolizumab (425)	Docetaxel (425)	330	520	0.64 (0.49-0.85)	0.79 (0.64-0.97)	No	Yes
25891173[71]	Robert *et al.* 2015	NCT01866319	Melanoma	Pembrolizumab	Ipilimumab	337	497	0.69 (0.46-1.04)	0.57 (0.39-0.84)	Yes	No
21639810[72]	Robert *et al.*2011	NCT00324155	Melanoma	Ipilimumab + dacarbazine (250)	Placebo + dacarbazine (252)	201	301	0.86 (0.63-1.17)	0.7 (0.55-0.90)	Yes	Yes
25399552[73]	Robert *et al.*2015	NCT01721772	Melanoma	Nivolumab (210)	Dacarbazine (208)	172	246	0.56 (0.33-0.95)	0.34 (0.22-0.54)	Yes	Yes

Abbreviation: F, female; M, male ccRCC, clear cell renal cell carcinoma; HNSC, head and neck cancer; NSCLC, non-small cell lung cancer; SCLC, small cell lung cancer; GOJC, Gastric or gastroesophageal junction carcinoma; MESO, Mesothelioma.

Supplementary Table 2. Molecular features between male and female patients with immune checkpoint blockade treatment

Dataset (PubMed ID/Refs)	Author and year	Cancer type	# F	# M	Drug class	Omics profiling	Biomarkers
29301960[9]	Miao *et al*. 2018	ccRCC	13	22	anti-PD-l	WXS	TMB, PBRM1 mutation
30643254[25]	Samstein *et al*. 2018	BLCA	51	163	anti-PD-l/PD-L1, Combo	WXS	TMB
		COAD	48	62	anti-PD-l/PD-L1, Combo, anti-CTLA4	WXS	TMB
		ESCA	28	98	anti-PD-l/PD-L1, Combo, anti-CTLA4	WXS	TMB
		GBM	40	77	anti-PD-l/PD-L1, Combo	WXS	TMB
		HNSC	30	108	anti-PD-l/PD-L1, Combo	WXS	TMB
		Melanoma	121	200	anti-PD-l/PD-L1,Combo, anti-CTLA4	WXS	TMB
		NSCLC	180	170	anti-PD-l/PD-L1, Combo	WXS	TMB
		ccRCC	42	109	anti-PD-l/PD-L1, Combo	WXS	TMB
30150660[74]	Miao *et al.* 2018	BLCA	8	19	anti-PD-l/PD-L1, Combo	WXS	TMB, neoantigen load, APOBEC
		NSCLC	33	24	anti-PD-l/PD-L1, Combo	WXS	TMB, neoantigen
		Melanoma	52	99	anti-PD-l/PD-L1, Combo, anti-CTLA4	WXS	TMB, neoantigen load, APOBEC
30309915[24]	Cristescu *et al.* 2018	HNSC	19	88	anti-PD-1	WXS, Nanostring	TMB, GEP
		Melanoma	31	58	anti-PD-1	WXS, Nanostring	TMB, GEP
		PanCan	67	52	anti-PD-1	WXS, Nanostring	TMB, GEP
26997480[21]	Hugo *et al.* 2016	Melanoma	11 (8*)	27 (19*)	anti-PD-1	WXS, RNA-seq	TMB, BRCA2 mutation
26359337[10]	Van Allen *et al.* 2015	Melanoma	32 (14*)	78 (28*)	anti-CTLA4	WXS, RNA-seq	TMB, neoantigen load, CYT, CTLA-4, PD-L2
29657128[22]	Hellmann *et al. 2018*	NSCLC	38	37	anti-PD-1 + anti-CTLA4	WXS	TMB, PD-L1

Abbreviations: F, female; M, male ccRCC, clear cell renal cell carcinoma; BLCA, bladder cancer; COAD, colorectal cancer; ESCA, esophagogastric cancer; GBM, glioma; HNSC, head and neck cancer; NSCLC, non-small cell lung cancer; PanCan, multiple cancer types with small sample size; TMB, tumor mutation burden; PD-L1/PD-L2,programmed cell death ligand 1/2; CTLA4, cytotoxic T-lymphocyte antigen-4; GEP, T cell–inflamed gene expression profile; APOBEC, apolipoprotein B mRNA editing enzyme, catalytic polypeptide-like; CYT, cytolytic activity.

Supplementary Table 3. Summary of patient samples and data types from TCGA analyzed in this study.

Cancer	Tumor mutation burden		Immune cell populations, checkpoint,CYT, and GEP		Protein expression		TCR, neoantigen load, and aneuploidy	
	F	M	F	M	F	M	F	M
ACC	44	28	45	29	NA	NA	44	29
BLCA	90	265	95	274	74	234	95	274
COAD	95	119	123	150	95	114	129	151
ESCA	26	158	26	158	NA	NA	25	157
GBM	43	93	45	93	20	39	47	96
HNSC	126	352	130	361	89	234	129	358
KICH	27	39	27	39	NA	NA	27	38
KIRC	141	254	165	297	128	259	162	284
KIRP	71	195	72	202	55	149	68	202
LGG	207	271	207	271	171	230	206	271
LIHC	120	247	121	250	69	112	121	247
LUAD	255	213	269	225	189	158	266	224
LUSC	44	126	124	354	74	235	121	344
PAAD	79	98	80	98	49	49	80	97
PCPG	101	78	101	78	40	39	101	77
READ	26	28	41	49	33	37	44	46
SARC	132	113	141	118	115	104	138	117
SKCM	33	47	42	61	42	53	45	63
STAD	121	220	127	253	100	210	128	252
THCA	346	128	351	133	247	106	349	131
THYM	56	63	57	63	41	46	57	63
UVM	35	45	35	45	NA	NA	35	45

Abbreviation: CYT, cytolytic activity; GEP, T cell–inflamed gene expression profile; TCR, T cell receptor; F, female; M, male; ACC, Adrenocortical carcinoma; BLCA, Bladder Urothelial Carcinoma; COAD, Colon adenocarcinoma; ESCA, Esophageal carcinoma; GBM, Glioblastoma multiforme; HNSC, Head and Neck squamous cell carcinoma; KICH, Kidney Chromophobe; KIRC, Kidney renal clear cell carcinoma; KIRP, Kidney renal papillary cell carcinoma; LGG, Brain Lower Grade Glioma; LIHC, Liver hepatocellular carcinoma; LUAD, Lung adenocarcinoma; LUSC, Lung squamous cell carcinoma; PAAD, Pancreatic adenocarcinoma; PCPG, Pheochromocytoma and Paraganglioma; READ, Rectum adenocarcinoma; SARC, Sarcoma; SKCM, Skin Cutaneous Melanoma; STAD, Stomach adenocarcinoma; THCA, Thyroid carcinoma; THYM, Thymoma; UVM, Uveal Melanoma

References

47. Chiappori, A. *et al.* Overall Survival with Durvalumab after Chemoradiotherapy in Stage III NSCLC. **379,** 24 (2018).
48. Barlesi, F. *et al.* Avelumab versus docetaxel in patients with platinum-treated advanced non-small-cell lung cancer (JAVELIN Lung 200): an open-label, randomised, phase 3 study. *The Lancet Oncology* **19,** 1468–1479 (2018).
49. Bellmunt, J. *et al.* Pembrolizumab as second-line therapy for advanced urothelial carcinoma. *New England Journal of Medicine* **376,** 1015–1026 (2017).
50. Borghaei, H. *et al.* Nivolumab versus Docetaxel in Advanced Nonsquamous Non–Small-Cell Lung Cancer. *New England Journal of Medicine* **373,** 1627–1639 (2015).
51. Brahmer, J. *et al.* Nivolumab versus docetaxel in advanced squamous-cell non-small-cell lung cancer. *New England Journal of Medicine* **373,** 123–135 (2015).
52. Carbone, D. P. *et al.* First-line nivolumab in stage IV or recurrent non-small-cell lung cancer. *New England Journal of Medicine* **376,** 2415–2426 (2017).
53. Cohen, E. E. W. *et al.* Pembrolizumab versus methotrexate, docetaxel, or cetuximab for recurrent or metastatic head-and-neck squamous cell carcinoma (KEYNOTE-040): a randomised, open-label, phase 3 study. *The Lancet* **393,** 156–167 (2019).
54. Ferris, R. L. *et al.* Nivolumab for recurrent squamous-cell carcinoma of the head and neck. *New England Journal of Medicine* **375,** 1856–1867 (2016).
55. Gandhi, L. *et al.* Pembrolizumab plus chemotherapy in metastatic non-small-cell lung cancer. *New England Journal of Medicine* **378,** 2078–2092 (2018).
56. Govindan, R. *et al.* Phase III Trial of Ipilimumab Combined With Paclitaxel and Carboplatin in Advanced Squamous Non – Small-Cell Lung Cancer. **35,** (2019).
57. Herbst, R. S. *et al.* Pembrolizumab versus docetaxel for previously treated , PD-L1-positive , advanced non-small-cell lung cancer (KEYNOTE-010): a randomised controlled trial. *The Lancet* **387,** 1540–50 (2016).
58. Hodi, F. S. *et al.* Combined nivolumab and ipilimumab versus ipilimumab alone in patients with advanced melanoma: 2-year overall survival outcomes in a multicentre, randomised, controlled, phase 2 trial. *The Lancet Oncology* **17,** 1558–1568 (2016).
59. Hodi, F. S. *et al.* Improved Survival With Ipilimumab in Patients With Metastatic Melanoma. *The New England journal of medicine* **363,** 711–23 (2005).
60. Horn, L. *et al.* First-line atezolizumab plus chemotherapy in extensive-stage small-cell lung cancer. *New England Journal of Medicine* **379,** 2220–2229 (2018).
61. Kang, Y. K. *et al.* Nivolumab in patients with advanced gastric or gastro-oesophageal junction cancer refractory to, or intolerant of, at least two previous chemotherapy regimens (ONO-4538-12, ATTRACTION-2): a randomised, double-blind, placebo-controlled, phase 3 trial. *The Lancet* **390,** 2461–2471 (2017).
62. Larkin, J. *et al.* Overall survival in patients with advanced melanoma who received nivolumab versus investigator's choice chemotherapy in CheckMate 037: A Randomized, Controlled, Open-Label Phase III Trial. *Journal of Clinical Oncology* **36,** 383–390 (2018).
63. Maio, M. *et al.* Tremelimumab as second-line or third-line treatment in relapsed malignant mesothelioma (DETERMINE): a multicentre, international, randomised,

double-blind, placebo-controlled phase 2b trial. *The Lancet Oncology* **18,** 1261–1273 (2017).
64. Motzer, R. J. *et al.* Nivolumab plus Ipilimumab versus Sunitinib in advanced renal-cell carcinoma. *New England Journal of Medicine* **378,** 1277–1290 (2018).
65. Motzer, R. J. *et al.* Nivolumab versus everolimus in advanced renal-cell carcinoma. *New England Journal of Medicine* **373,** 1803–1813 (2015).
66. Paz-Ares, L. *et al.* Pembrolizumab plus chemotherapy for squamous non-small-cell lung cancer. *New England Journal of Medicine* **379,** 2040–2051 (2018).
67. Reck, M. *et al.* Phase III randomized trial of ipilimumab plus etoposide and platinum versus placebo plus etoposide and platinum in extensive-stage small-cell lung cancer. *Journal of Clinical Oncology* **34,** 3740–3748 (2016).
68. Reck, M. *et al.* Pembrolizumab versus Chemotherapy for PD-L1-Positive Non-Small-Cell Lung Cancer. *New England Journal of Medicine* **375,** 1823–1833 (2016).
69. Ribas, A. *et al.* Phase III randomized clinical trial comparing tremelimumab with standard-of-care chemotherapy in patients with advanced melanoma. *Journal of Clinical Oncology* **31,** 616–622 (2013).
70. Rittmeyer, A. *et al.* Atezolizumab versus docetaxel in patients with previously treated non-small-cell lung cancer (OAK): a phase 3, open-label, multicentre randomised controlled trial. *The Lancet* **389,** 255–265 (2017).
71. Robert, C. *et al.* Pembrolizumab versus ipilimumab in advanced melanoma. *New England Journal of Medicine* **372,** 2521–2532 (2015).
72. Robert, C. *et al.* Ipilimumab plus dacarbazine for previously untreated metastatic melanoma. *New England Journal of Medicine* **364,** 2517–2526 (2011).
73. Robert, C. *et al.* Nivolumab in previously untreated melanoma without BRAF mutation. *New England Journal of Medicine* **372,** 320–330 (2015).
74. Miao, D. *et al.* Genomic correlates of response to immune checkpoint blockade in microsatellite-stable solid tumors. *Nature Genetics* **50,** 1271–1281 (2018).
