# Supplementary material for: Sex-associated molecular differences for cancer immunotherapy
Source: Nat Commun. 2020 Apr 14;11:1779. doi: 10.1038/s41467-020-15679-x (PMC7156379; doi:10.1038/s41467-020-15679-x)
Supplement: Supplementary file 3 — Reporting Summary [file 41467_2020_15679_MOESM3_ESM.pdf]

nature research

Corresponding author(s): Leng Han

Last updated by author(s): 2019-3-9

Reporting Summary

Nature Research wishes to improve the reproducibility of the work that we publish. This form provides structure for consistency and transparency in reporting. For further information on Nature Research policies, see Authors & Referees and the Editorial Policy Checklist .

Statistics

For all statistical analyses, confirm that the following items are present in the figure legend, table legend, main text, or Methods section.

n/a	Confirmed	
☐	☒	The exact sample size (*n*) for each experimental group/condition, given as a discrete number and unit of measurement
☒	☐	A statement on whether measurements were taken from distinct samples or whether the same sample was measured repeatedly
☐	☒	The statistical test(s) used AND whether they are one- or two-sided<br>*Only common tests should be described solely by name; describe more complex techniques in the Methods section.*
☐	☒	A description of all covariates tested
☐	☒	A description of any assumptions or corrections, such as tests of normality and adjustment for multiple comparisons
☐	☒	A full description of the statistical parameters including central tendency (e.g. means) or other basic estimates (e.g. regression coefficient) AND variation (e.g. standard deviation) or associated estimates of uncertainty (e.g. confidence intervals)
☐	☒	For null hypothesis testing, the test statistic (e.g. *F*, *t*, *r*) with confidence intervals, effect sizes, degrees of freedom and *P* value noted<br>*Give P values as exact values whenever suitable.*
☒	☐	For Bayesian analysis, information on the choice of priors and Markov chain Monte Carlo settings
☒	☐	For hierarchical and complex designs, identification of the appropriate level for tests and full reporting of outcomes
☒	☐	Estimates of effect sizes (e.g. Cohen's *d*, Pearson's *r*), indicating how they were calculated

Our web collection on statistics for biologists contains articles on many of the points above.

Software and code

Policy information about availability of computer code

Data collection	No software was used for data collection.
Data analysis	All data analysis was performed by R programming (R 3.6.2). R codes are deposited in GitHub: https://github.com/youqiongye/SexImm.

For manuscripts utilizing custom algorithms or software that are central to the research but not yet described in published literature, software must be made available to editors/reviewers. We strongly encourage code deposition in a community repository (e.g. GitHub). See the Nature Research guidelines for submitting code & software for further information.

Data

Policy information about availability of data

All manuscripts must include a data availability statement. This statement should provide the following information, where applicable:

- Accession codes, unique identifiers, or web links for publicly available datasets
- A list of figures that have associated raw data
- A description of any restrictions on data availability

All data supporting the findings of the current study are listed in the Supplementary Tables 1-3. All associated raw data are available in Supplementary Data 1 and 2 for Figs. 1-4 and Supplementary Figs. 1-8, respectively. TCGA dataset and all other related public datasets have been referenced in the manuscript.

Field-specific reporting

Please select the one below that is the best fit for your research. If you are not sure, read the appropriate sections before making your selection.

☒ Life sciences ☐ Behavioural & social sciences ☐ Ecological, evolutionary & environmental sciences

Life sciences study design

All studies must disclose on these points even when the disclosure is negative.

Sample size	1. In immune checkpoint blockade (ICB) treatment trials, there are 11313 male and 5711 female cancer patients. The detailed sample size for male and female patients in each clinical trial was listed in Supplementary Table 1.<br>2. In ICB treatment datasets with molecular profiling for individual patients, there are 1491 male and 844 female cancer patients. The detailed sample size for male and female patients in each dataset was listed in Supplementary Table 2.<br>3. In TCGA pan-cancer analysis, we analyzed all tumors with multi-omics data and clinical data from TCGA data portal (https://tcga-data.nci.nih.gov/tcga/) (n = 9686). The detailed sample size for male and female patients in each cancer was listed in Supplementary Table 3.<br>We described sample size for all analyses clearly in the methods section and supplementary files.
Data exclusions	We kept 22 cancer types with ≥20 samples in both female and male groups and didn't further exclude any data.
Replication	All data analyses are reproducible. All codes are carefully checked to ensure the replication.
Randomization	All data was obtained from public data resources, so randomization is not relevant to our study.
Blinding	All data was obtained from public data resources, so blinding is not relevant to our study.

Reporting for specific materials, systems and methods

We require information from authors about some types of materials, experimental systems and methods used in many studies. Here, indicate whether each material, system or method listed is relevant to your study. If you are not sure if a list item applies to your research, read the appropriate section before selecting a response.

Materials & experimental systems

n/a	Involved in the study
☒	☐ Antibodies
☒	☐ Eukaryotic cell lines
☒	☐ Palaeontology
☒	☐ Animals and other organisms
☒	☐ Human research participants
☒	☐ Clinical data

Methods

n/a	Involved in the study
☒	☐ ChIP-seq
☒	☐ Flow cytometry
☒	☐ MRI-based neuroimaging
